# Identification of AKI signatures and classification patterns in ccRCC based on machine learning

**DOI:** 10.3389/fmed.2023.1195678

**Published:** 2023-05-24

**Authors:** Li Wang, Fei Peng, Zhen Hua Li, Yu Fei Deng, Meng Na Ruan, Zhi Guo Mao, Lin Li

**Affiliations:** ^1^Department of Nephrology, Changzheng Hospital, Naval Medical University, Shanghai, China; ^2^Department of Cardiology, Jinshan Hospital of Fudan University, Shanghai, China; ^3^Department of Cardiology, Changzheng Hospital, Naval Medical University, Shanghai, China

**Keywords:** acute kidney injury, machine learning, molecular subtypes, immunity, clear cell renal cell carcinoma

## Abstract

**Background:**

Acute kidney injury can be mitigated if detected early. There are limited biomarkers for predicting acute kidney injury (AKI). In this study, we used public databases with machine learning algorithms to identify novel biomarkers to predict AKI. In addition, the interaction between AKI and clear cell renal cell carcinoma (ccRCC) remain elusive.

**Methods:**

Four public AKI datasets (GSE126805, GSE139061, GSE30718, and GSE90861) treated as discovery datasets and one (GSE43974) treated as a validation dataset were downloaded from the Gene Expression Omnibus (GEO) database. Differentially expressed genes (DEGs) between AKI and normal kidney tissues were identified using the R package limma. Four machine learning algorithms were used to identify the novel AKI biomarkers. The correlations between the seven biomarkers and immune cells or their components were calculated using the R package ggcor. Furthermore, two distinct ccRCC subtypes with different prognoses and immune characteristics were identified and verified using seven novel biomarkers.

**Results:**

Seven robust AKI signatures were identified using the four machine learning methods. The immune infiltration analysis revealed that the numbers of activated CD4 T cells, CD56^dim^ natural killer cells, eosinophils, mast cells, memory B cells, natural killer T cells, neutrophils, T follicular helper cells, and type 1 T helper cells were significantly higher in the AKI cluster. The nomogram for prediction of AKI risk demonstrated satisfactory discrimination with an Area Under the Curve (AUC) of 0.919 in the training set and 0.945 in the testing set. In addition, the calibration plot demonstrated few errors between the predicted and actual values. In a separate analysis, the immune components and cellular differences between the two ccRCC subtypes based on their AKI signatures were compared. Patients in the CS1 had better overall survival, progression-free survival, drug sensitivity, and survival probability.

**Conclusion:**

Our study identified seven distinct AKI-related biomarkers based on four machine learning methods and proposed a nomogram for stratified AKI risk prediction. We also confirmed that AKI signatures were valuable for predicting ccRCC prognosis. The current work not only sheds light on the early prediction of AKI, but also provides new insights into the correlation between AKI and ccRCC.

## 1. Introduction

Acute kidney injury (AKI) is a complex clinical disorder that manifests as a rapid decrease in glomerular filtration rate within three months and an increase in serum creatinine levels (1). Several stimuli may contribute to the development of AKI. Ischemia–reperfusion injury is one of the major clinical challenges faced by clinicians, especially during the perioperative period of renal transplantation. In AKI patients, persistent renal dysfunction and irreversible nephron loss can lead to chronic kidney failure. In addition, the risk of cardiovascular problems and other complications increases over time in renal failure patients (2), and critically ill patients have even worse prognoses (3). Therefore, the prevention and early detection of AKI are of paramount importance, and it is essential to identify specific signatures in patients with AKI. However, although biomarkers for the detection of AKI such as cystatin C, liver-type fatty acid-binding protein, interleukin-18, and neutrophil gelatinase-associated lipocalin have begun to emerge, these have some limitations (4). A useful tool related to this type of research is machine learning, a field of artificial intelligence that uses computer systems to process data using complex mathematical algorithms. With its powerful algorithms, machine learning has recently been applied in the field of medicine (5, 6). However, few studies have used machine learning to identify novel AKI biomarkers.

Several lines of evidence suggest that AKI induces or promotes the occurrence and progression of renal cancer. For instance, kidney injury may trigger DNA damage and promote mutated cell clonal proliferation in different kidney compartments (7). In one study, Peired et al. demonstrated a correlation between AKI and the subsequent development of renal cancer by analyzing patients with AKI from multiple independent cohorts (8). In addition, previous studies have shown that renal cancer progression is based on the increased systemic production of various chemokines, cytokines, and inflammatory immune cells (9–11). In another study, Zhou et al. demonstrated that AKI induces a systemic inflammatory response through modulation by inhibitors of the CXCL1/CXCR2 axis, thus increasing the risk of clear cell renal cell carcinoma (ccRCC) formation (12). Moreover, several studies have suggested that renal carcinoma occurs after an AKI episode or after years of chronic kidney disease, as kidney injury is an initial factor in renal cancer (8, 13). Indeed, ccRCCs account for approximately 75% of renal carcinoma cases (14). However, there is no satisfactory AKI-based prognostic model for accurate risk stratification in these patients. Therefore, it is essential to identify specific classifications for ccRCC prognostic prediction at the AKI level.

In this study, public datasets on AKI and ccRCC were downloaded and analyzed. We identified AKI-related biomarkers using functional pathway analysis, immune analysis, and AKI risk prediction. In addition, we carried out an AKI-related signature study by classifying ccRCC patients and integrating biological function enrichment, immune infiltration, chemical drug sensitivity data, and prognostic analyses. Furthermore, reliable signature subtyping was performed to predict the prognosis of patients with ccRCC based on the crucial roles of novel AKI biomarkers. Notably, this is the first study to elucidate novel AKI-related biomarkers with prognostic predictive value in ccRCC.

## 2. Materials and methods

### 2.1. Data collection and processing

Five public AKI datasets from the Gene Expression Omnibus (GEO) were downloaded and processed: GSE126805 (n = 83; normal tissues = 41, AKI tissues = 42), GSE139061 (n = 48; normal tissues = 9, AKI tissues = 39), GSE30718 (*n* = 47; normal tissues = 19, AKI tissues = 28), GSE90861 (*n* = 46; normal tissues = 23, AKI tissues = 23), and GSE43974 (*n* = 391; normal tissues = 188, AKI tissues = 203). The former four datasets were integrated and the batch was removed to construct a training cohort, whereas GSE43974 was treated as an independent test cohort. GSE126805, GSE139061, GSE30718, and GSE90861 were used to test whether these biomarkers could distinguish AKI tissues from normal tissues. Approval and informed consent from the institutional review board were not required for the AKI cohorts from the public databases. To reveal the connection between AKI and ccRCC, ccRCC multi-omics information was retrieved from the GDC TCGA portal.

### 2.2. Batch effect removal

To remove batch effects derived from the study design, sequence platform, and technological replication, we filtered only normal and AKI tissue expression matrices and clinical characteristics from four cohorts: GSE126805, GSE139061, GSE30718, and GSE90861. The batch effect was removed using the default function in the sva package. A principal component analysis (PCA) was used to visualize the efficacy of the batch removal.

### 2.3. Differential expression and enrichment analysis

We used the limma package to identify differentially expressed genes (DEGs) between normal and AKI tissues in the merged expression matrix. DEGs were identified using a significance threshold of *p* < 0.05 and an absolute log-fold change >1.5. An enrichment analysis of the DEGs was performed using the clusterProfiler and ggpplot2 packages, and these were visualized using an enrichment plot. Additionally, Gene Ontology (GO), Kyoto Encyclopedia of Genes and Genomes (KEGG), and gene set enrichment analyses (GSEA) were performed to better understand the biological roles of these DEGs. The significantly differentially expressed terms or pathways were identified by a threshold value of *p* <0.05 and *q*-value <0.05.

### 2.4. Identification and verification of AKI-related biomarkers

After identifying the DEGs from the combined expression profile of the combined expression matrix, we sought to identify the most relevant AKI-related biomarkers. We adopted four machine-learning algorithms, including Least Absolute Shrinkage and Selection Operator (LASSO) logistic regression, Random Forest (RF), eXtreme Gradient Boosting (XGBoost), and support vector machine (SVM), to select reliable biomarkers to distinguish AKI from normal tissues. The detailed parameters adopted for these were as recommended in each of the R packages. The detailed parameters of LASSO logistics were as follow: alpha = 1, maximum number of iterations = 8,000, tol = 1e-4). The detailed parameters of Random Forest were as follow: n_estimators = 80, criterion = gini, min_samples_split = 2, min_samples_leaf = 1. And for XGBoost, the detailed hyperparameters were as follow: n_estimators = 500, max_depth = 6, reg_alpha = 0, colsample_bytree = 1. Finally, the detailed parameters of SVM were as follow: tol = 1e-3, max_iter = −1. Furthermore, the receiver operating characteristic (ROC) curve was adopted to further evaluate the accuracy of the filtered biomarkers in the training and testing cohorts using the R package pROC. The GSE90861 dataset was used as a validation cohort to verify the specificity and sensitivity of the biomarkers retrieved from the test cohort.

### 2.5. Immune components and cell differences between AKI and normal tissues

Single-sample gene set enrichment analysis (ssGSEA) and bulk sequence-based deconvolution algorithms from the GSVA and ESTIMATE packages were used to detect different immune cells and components between normal and AKI tissues. The gene sets used for ssGSEA consisted of 28 types of immune cells, including activated B cells, activated CD4 T cells, activated CD8 T cells, activated dendritic cells, CD56^bright^ natural killer cells, CD56^dim^ natural killer cells, central memory CD4 T cells, central memory CD8 T cells, effector memory CD4 T cells, effector memory CD8 T cells, eosinophils, gamma delta T cells, immature B cells, immature dendritic cells, macrophages, mast cells, MDSCs, memory B cells, monocytes, natural killer cells, natural killer T cells, neutrophils, plasmacytoid dendritic cells, regulatory T cells, T follicular helper cells, type 1 T helper cells, type 17 T helper cells, and type 2 T helper cells. The estimated algorithms contained three scores: ESTIMATE, immune, and stromal scores. The correlations between the seven biomarkers and immune cells or their components were calculated using the R package ggcor.

### 2.6. Identification of different subtypes of AKI and ccRCC

To further reveal the heterogeneity of AKI and the potential relationship between AKI and ccRCC, we performed an unsupervised cluster analysis based on seven novel biomarkers (*CCNL1*, *NFKBIZ*, *HBB*, *TRIB1*, *SOCS3*, *HSPA6*, and *EGR1*) using the Consensus Cluster Plus package. The optimal cluster number was identified based on the PCA algorithm and cumulative distribution function curve. Differences in estimates, immune scores, and immune components were also qualified and compared between the ccRCC subtypes. A detailed description of the parameters employed are described in previous studies (15, 16).

## 3. Results

### 3.1. Batch effects removal and data integration

**Figure 1:**
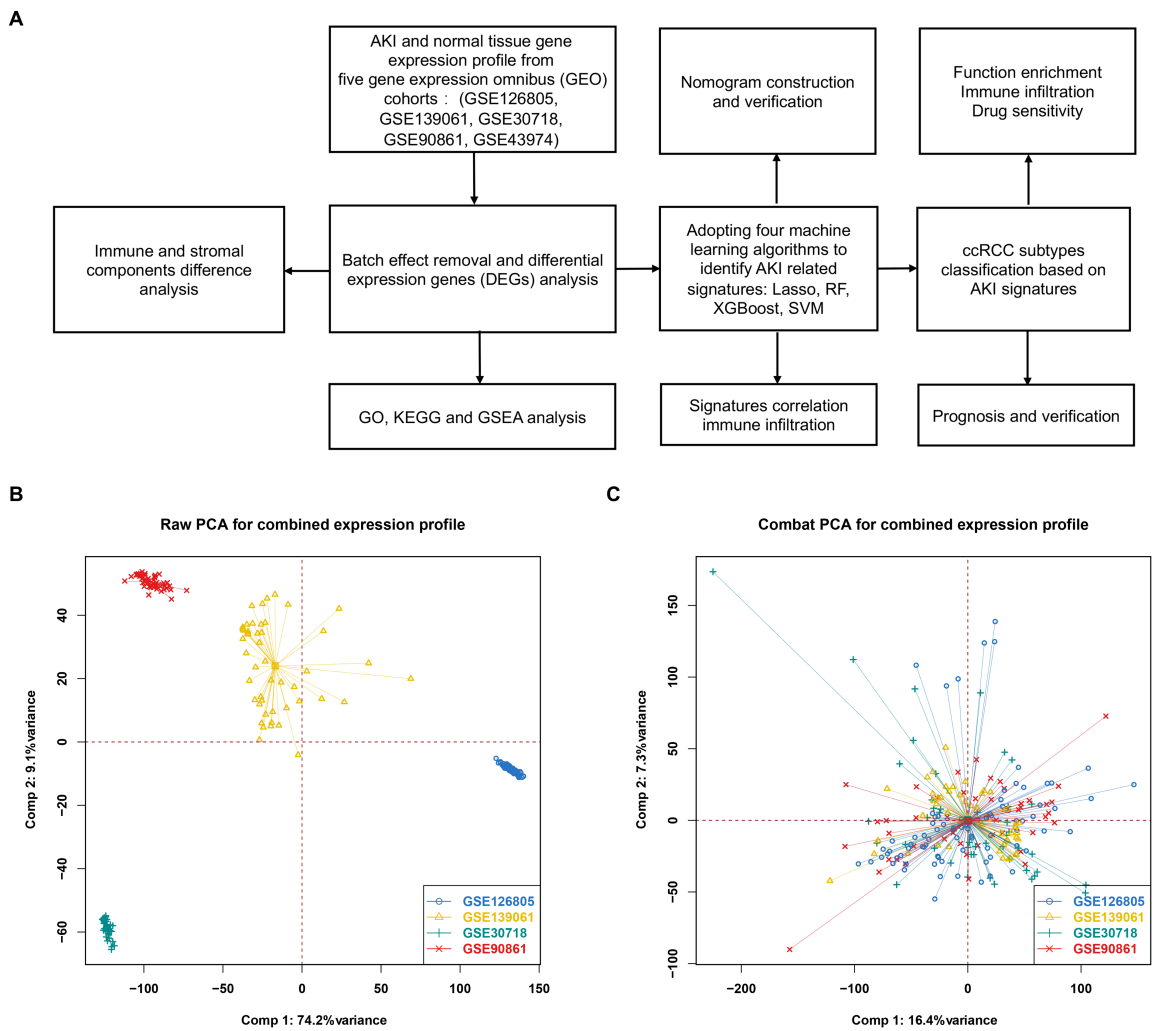
Differentially expressed genes between AKI and normal tissues. **(A)** Workflow of our study. **(B)** PCA plot before batch effect removal. **(C)** PCA after batch effect removal. PCA, principal component analysis.

Figure 1A shows a flowchart of our study. Figure 1B shows the raw PCA results for the four microarray databases (GSE126805, GSE139061, GSE30718, and GSE90861). The four different colors represent different datasets. Every dataset was discrete without any intersection. Figure 1C shows the combat PCA results following batch removal.

### 3.2. Functional pathway enrichment analysis

**Figure 2:**
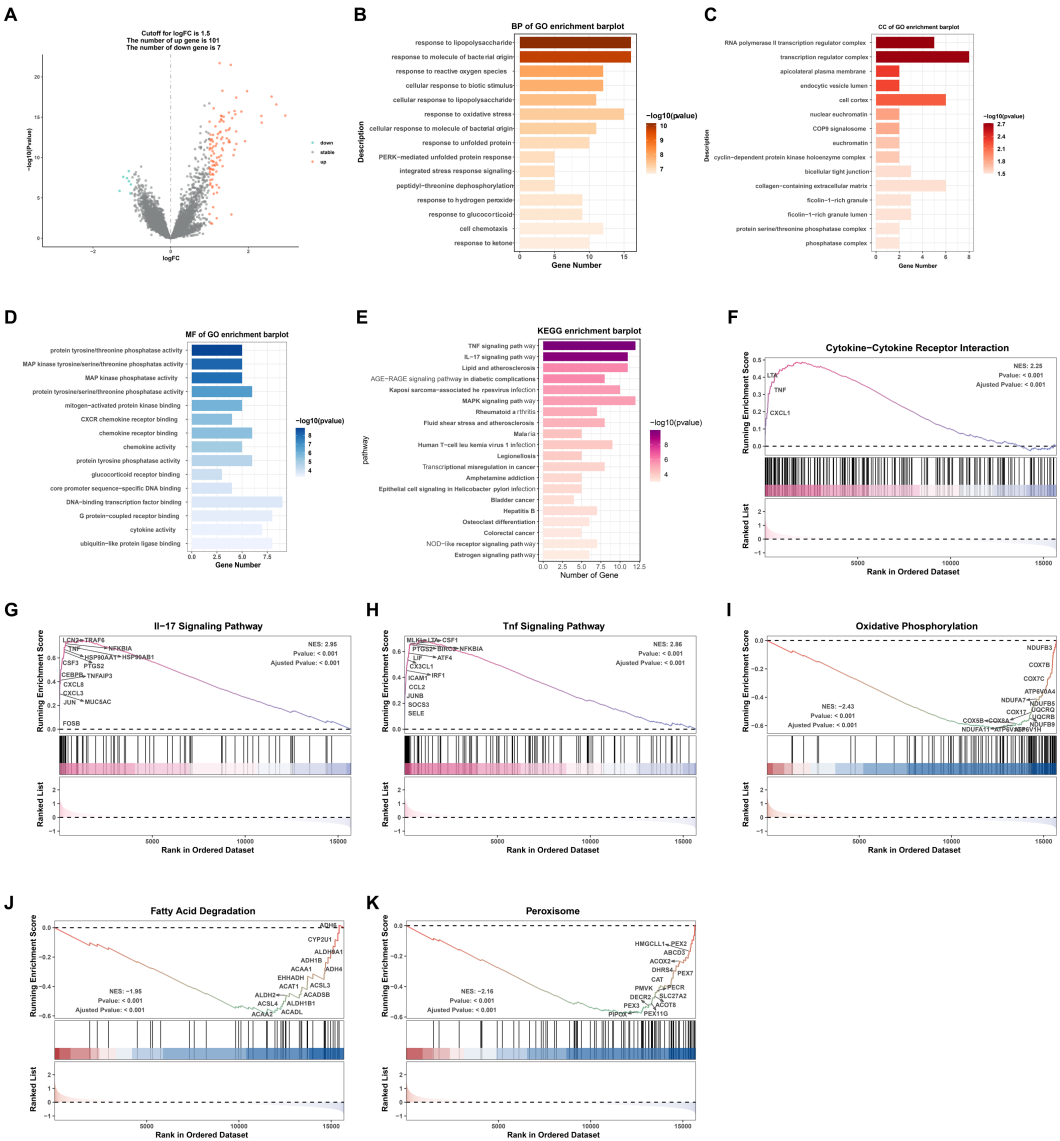
GO/KEGG/GSEA analysis of common differentially expressed genes (DEGs). **(A)** Volcano plot of the DEGs. **(B)** BP identified through GO analysis of the DEGs. **(C)** CC identified through GO analysis of the DEGs. **(D)** MF identified through GO analysis of the DEGs. **(E)** KEGG analysis of the differentially expressed signatures. **(F–K)** GSEA plot showing the most enriched gene sets based on KEGG analysis of all detected genes. BP, biological process; CC, cellular component; MF, molecular function; GO, Gene Ontology; KEGG, Kyoto Encyclopedia of Genes and Genomes; GSEA, gene set enrichment analysis; FC, fold change.

A volcano map (Figure 2A) was created to visually display the expression changes of the DEGs between normal and AKI tissues. A total of 108 genes were identified as DEGs according to adjusted *p*-values <0.05 and logFC cutoffs >1.5; among these, 101 genes were upregulated and 7 genes were downregulated. Next, we performed GO and KEGG enrichment analyses. As depicted in Figures 2B–D, in terms of biological processes (BP), the DEGs were mostly associated with lipopolysaccharides and molecules of bacterial origin. Regarding cellular components (CC), these were mainly involved in the RNA polymerase II transcription regulator complex and transcription regulator complex. In terms of molecular functions (MF), the DEGs were largely associated with tyrosine proteins, threonine phosphatase activity, and MAP kinases. The 20 most significant KEGG pathway terms are shown in Figure 2E. These genes were primarily associated with the TNF signaling pathway, the IL-17 signaling pathway, lipid and atherosclerosis pathways, the AGE − RAGE signaling pathway in those with diabetic complications, and the MAPK signaling pathway.

### 3.3. GSEA analysis of the AKI and normal groups

GSEA was performed to determine which KEGG pathways were differentially enriched between the AKI and normal groups according to the NES and value of p (*p* < 0.01) criteria. As presented in Figures 2F–K, the top three significantly upregulated KEGG pathways enriched in the AKI group were pathways related to cytokine-cytokine receptor interactions, IL-17 signaling, and TNF signaling. In addition, oxidative phosphorylation, fatty acid degradation, and peroxisomes were significantly downregulated in the AKI tissues. These data reveal that numerous pathways may be directly or indirectly involved in the regulation of AKI.

### 3.4. AKI signature identification and validation

**Figure 3:**
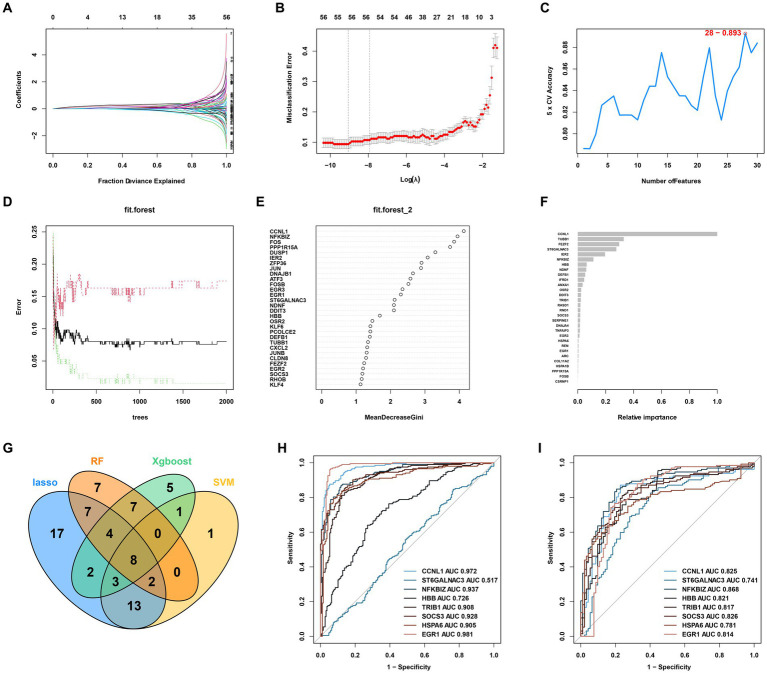
AKI signature identification and verification. **(A,B)** LASSO. **(C)** SVM. **(D,E)** RF 2. **(F)** XGBoost. **(G)** Venn diagram for the four machine learning algorithms. **(H)** ROC curves for the eight genes in the training cohorts. **(I)** Testing cohorts. LASSO, Least Absolute Shrinkage and Selection Operator; SVM, support vector machine; RF, Random Forest; XGBoost, Xtreme Gradient Boosting; ROC, receiver operating characteristic.

**Figure 4:**
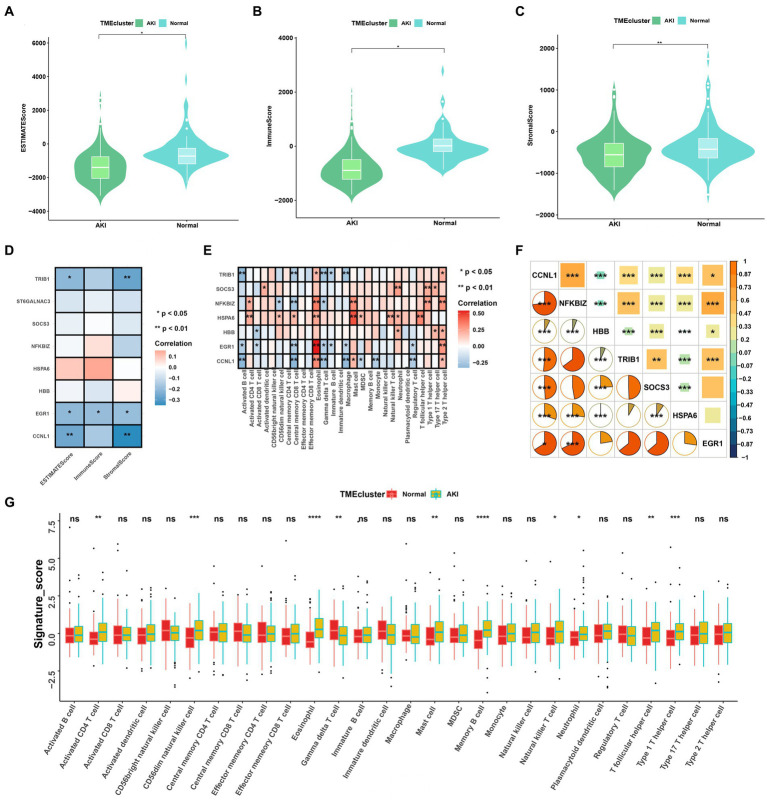
Immune cell infiltration rates and the correlations among these and the signatures. **(A–C)** ESTIMATE, immune, and stromal score differences between the ccRCC subtypes. **(D)** Correlations between ESTIMATE scores and significant genes. **(E)** Enriched signatures in the immune cells. **(F)** Correlations among the seven signatures according to Pearson and Spearman analyses. **(G)** Boxplot of immune cell infiltration between the normal and AKI clusters. ns > 0.05, *p < 0.05, **p < 0.01, ***p < 0.001, ns, not significant.

Four machine learning algorithms were applied to identify feature genes: LASSO regression (Figures 3A,B), SVM (Figure 3C), and Random Forest (Figures 3D,E). These were combined with a feature selection analysis to determine the top 30 genes with relative importance, and an XGBoost analysis was then performed using these 30 relatively important genes (Figure 3F). A Venn diagram was used to display the overlapping signatures of the four methods (Figure 4G). After applying the four machine learning algorithms, eight signatures were selected, as shown in Figures 3A–G: tribbles homolog 1 (*TRIB1*), *ST6GALNAC3*, suppressors of cytokine signaling 3 (*SOCS3*), NF-κB inhibitor ζ (*NFKBIZ*), heat shock protein family A 6 (*HSPA6*), hemoglobin subunit beta (*HBB*), early growth response 1 (*EGR1*), and cyclin L1 (*CCNL1*).

As shown in Figures 3H,I, to further test the diagnostic efficacy of the above genes, ROC analysis was performed to evaluate the accuracy of each gene. The ROC curve showed that the seven biomarkers identified based on the logistic regression were reliable, with the following AUCs in the training set: *CCNL1*, 0.972; *NFKBIZ*, 0.937; *HBB*, 0.726; *TRIB1*, 0.908; *SOCS3*, 0.928; *HSPA6*, 0.905; and *EGR1*, 0.981. In the validation set, the AUCs were as follows: *CCNL1*, 0.825; *NFKBIZ*, 0.868; *HBB*, 0.821; *TRIB1*, 0.817; *SOCS3*, 0.826; *HSPA6*, 0.781; and *EGR1*, 0.814. *ST6GALNAC3* (AUC = 0.517 in the test set) was not analyzed further in the next workflow.

### 3.5. AKI marker genes and immune cell infiltration

We analyzed the heterogeneous compositions of the tumor microenvironments (TMEs) in the AKI and normal groups. As depicted in Figures 4A–C, all estimated scores, including the immune, stromal, and estimated scores, were lower in the AKI group compared to the normal group. We then examined the specific correlations between the estimated scores and each of the signatures using Spearman’s correlation analyses, which revealed that *TRIB1*, *EGR1*, and *CCNL1* were related to the estimate and stromal scores (Figure 4D). As shown in Figure 4E, almost all of these genes were positively or negatively correlated with immune cell infiltration. Of these, *EGR1* and eosinophil, *HSPA6* and mast cells were significantly and positively relevant. In contrast, *TRIB1* and activated B cells, *CCNL1* and central memory CD8 T cells were negatively correlated (*p* < 0.01).

As shown in Figure 4F, the hub gene correlation analysis revealed that the expression levels of *CCNL1* and *NFKBIZ* were mostly positively correlated, and *CCNL1* was linked to every other gene. Figure 4G shows that 28 immune cells were differentially concentrated between the AKI and control groups. Among these, activated CD4 T cells, CD56^dim^ natural killer cells, eosinophils, mast cells, memory B cells, natural killer T cells, neutrophils, T follicular helper cells, and type 1 T helper cells were more enriched in the AKI cluster.

### 3.6. Construction of the nomogram model

**Figure 5:**
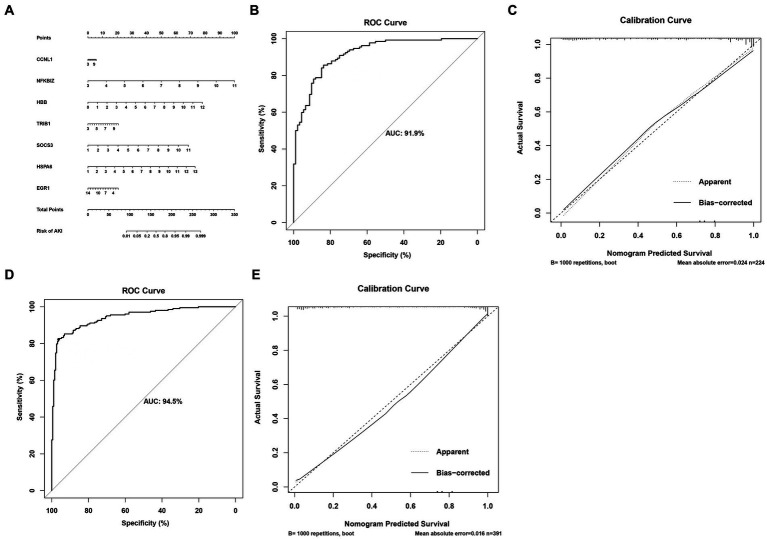
Nomogram for predicting the risk of AKI. **(A)** Nomogram. **(B,C)** ROC curves for the sensitivity of the nomogram and the calibration curves in the training set. **(D,E)** ROC curves and calibration curves in the validation set.

In order to accurately predict AKI risk using these signatures, a nomogram was constructed. As shown in Figure 5A, in the nomogram, each biomarker gene had a parallel score within the range of 0–100 points in terms of its association with the risk of AKI. As shown in Figures 5B–E, the AUC value was 0.919 in the training set and 0.945 in the testing set. In both the training and validation sets, the calibration plot demonstrated a good fit of the constructed nomogram, indicating satisfactory model accuracy.

### 3.7. ccRCC subtypes with the AKI-related gene classification

**Figure 6:**
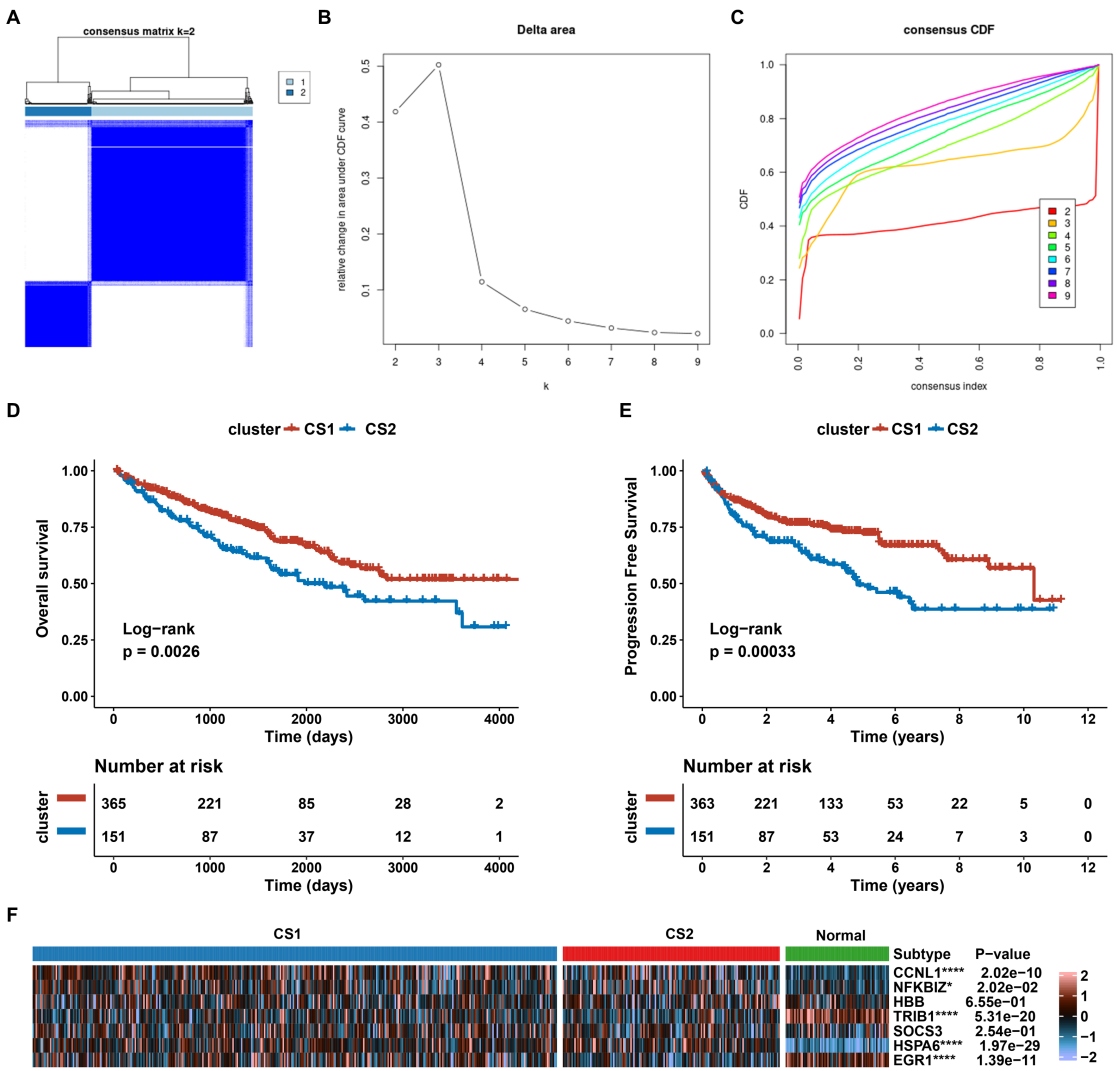
Establishment of two clusters based on AKI signatures in ccRCC. **(A)** Consensus cluster matrix of TCGA-ccRCC samples when *k* = 2. **(B)** Relative change of delta area under different cluster number. **(C)** Cumulative distribution function curves, *k* = 2 to 9. **(D,E)** Survival analysis for overall survival (OS) and progression-free survival (PFS) among the two subtypes in the TCGA-ccRCC dataset. **(F)** Expression profiles of AKI biomarker genes among the two subtypes and normal tissues. ns > 0.05, *p < 0.05, **p < 0.01, ***p < 0.001, ****p < 0.0001. ns, not significant.

We investigated the expression patterns of AKI-related signatures in ccRCC to comprehensively explore the association between AKI and cancer. TCGA-ccRCC samples were classified into different molecular subtypes according to AKI signature expression levels. For this, we divided the optimal cluster numbers using an unsupervised clustering method. As depicted in Figures 6A–C, considering the relative change in area under cumulative distribution function curve and consensus index, we chose k = 2 as the optimal cluster number. The TCGA ccRCC dataset was classified into two optimal clusters: CS1 and CS2. Compared to CS2 patients, CS1 patients had better overall survival (OS) (*p* = 0.0026, log-rank test) and progression-free survival (PFS) (*p* = 0.00033, log-rank test) (Figures 6D,E). In addition, we analyzed the expression levels of AKI genes in normal tissues and in the two ccRCC subtypes using a heatmap. *CCNL1*, *NFKBIZ*, *SOCS3*, and *HSPA6* were significantly upregulated in the ccRCC subtype compared to normal tissues, whereas *TRIB1* and *EGR1* were significantly upregulated in the ccRCC subtypes (*p* < 0.05; Figure 6F).

### 3.8. Gene expression profiles of the ccRCC subtypes and results of the function enrichment analysis

Considering the varying characteristics of each cluster, we identified the gene expression profiles of CS1 and CS2. Supplementary Figure S1A presents an enhanced volcano map that displays the differential gene expression between CS1 and CS2, with 19,168 total differentially expressed genes. In addition, the GO analysis demonstrated that DEGs between ccRCC subtypes were annotated in receptor antagonist, receptor inhibitor, peptidase inhibitor, peptidase regulator, and enzyme inhibitor activity in biological process (Supplementary Figure S1B).

Supplementary Figure S1C shows the results of the GSVA algorithm analysis, which demonstrates that G2M-checkpoint, TGFβ signaling, and E2F oncogenes were more enriched in CS2 than in CS1. In addition, regulon analysis was used to detect transcriptome differences, which demonstrated that *HNF1A*, *EPAS1*, *ZEB2*, *TFE3*, and *TP53* were downregulated in CS2, whereas *FOXE1* and *TBX18* were upregulated in CS1 (Supplementary Figure S1D).

### 3.9. Comparison of immune infiltration between the two ccRCC subtypes

**Figure 7:**
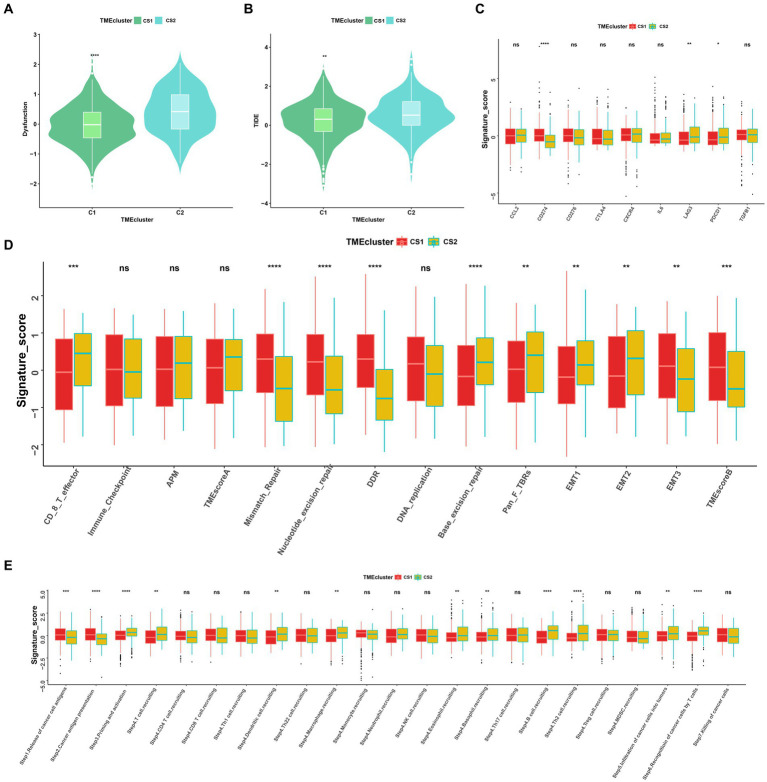
Landscapes of specific immune function scores and immune components. **(A,B)** Immune function scores (Dysfunction and TIDE) among the two ccRCC subtypes. **(C–E)** Immune antigens, immune pathways, and anti-cancer steps among the two subtypes. ns > 0.05, *p < 0.05, **p < 0.01, ***p < 0.001, ****p < 0.0001. ns, not significant.

To further elucidate the molecular distinctions between the two ccRCC subtypes, we conducted a comprehensive analysis of the immune-related signatures, signaling pathways, anticancer responses, and other relevant factors in both subtypes. We found that the dysfunction scores and Tumor Immune Dysfunction and Exclusion (TIDE) scores in CS1 were significantly lower than those in CS2 (Figures 7A,B), which indicated lower tumor immune evasion and resistance to checkpoint blockage in this cluster. In addition, the expression levels of nine immune checkpoint inhibitor genes were compared between the CS1 and CS2 cells; among them, *CD274* was significantly upregulated in CS1 cells, whereas *LAG3* and *PDCD1* were significantly downregulated in CS2 cells (Figure 7C).

Among the different immune-related signatures expressed between the two ccRCC subtypes in the heatmap (Supplementary Figure S2A), chemokines, chemokine receptors, major histocompatibility complexes, and immunoinhibitory and immunostimulatory signatures were significantly differentially expressed. The CS1 subtype showed lower expression of *CXCL11*, *CCR4*, *HLA-DOA*, *CD244*, and *IL2RA*. Moreover, CS1 showed higher immune cell infiltration than CS2 in the TME infiltration cell-type heatmap (Supplementary Figure S2B). Mismatch repair, nucleotide excision repair, and base excision repair signatures were significantly higher in CS1, whereas EMT1, EMT2, signatures were significantly lower this cluster (Figure 7D).

As the anticancer immune response can be interpreted as a series of immune cell combat processes, we evaluated the tumor immunophenotypes in the two clusters. As shown in Figure 7E, the CS2 cluster demonstrated higher activity associated with priming and activation (step3), T cell recruiting (step4), dendritic cell recruiting (step4), macrophage recruiting (step4), B cell recruiting (step4), Th2 cell recruiting (step4) and recognition of cancer cells by T cell (step6). In contrast, CS1 demonstrated higher activity associated with the release of cancer antigen (step1) and cancer antigen presentation activity (step2) (*p* < 0.05).

### 3.10. Drug sensitivity and genomic mutation analysis of the two subtypes

**Figure 8:**
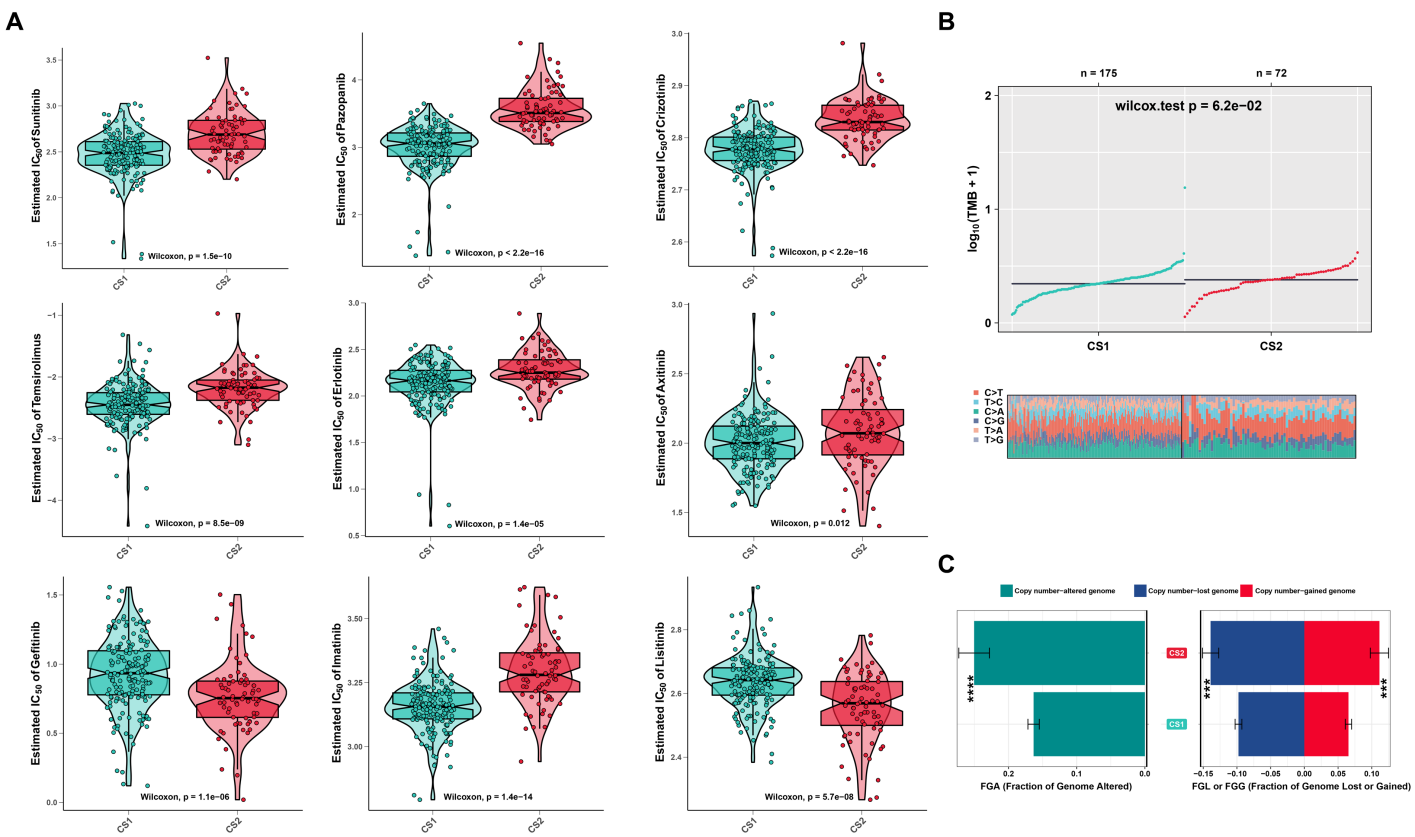
Drug sensitivity analysis and other omics of the two ccRCC subtypes. **(A)** Estimated IC50 of the indicated molecular-targeted drugs among the two ccRCC subtypes. **(B)** Tumor mutation burden between the two subtypes. **(C)** Bar plot of the genomic fractions that were altered between the two subtypes. ns > 0.05, *p < 0.05, **p < 0.01, ***p < 0.001, ****p < 0.0001. ns, not significant.

To evaluate the drug responses of the ccRCC subtypes, the estimated IC50 data for each drug were collected from the GDSC database to explore the potential chemotherapy sensitivity of ccRCC. We found that the CS1 subtype was more sensitive to sunitinib, pazopanib, crizotinib, erlotinib, temsirolimus, and axitinib, whereas the CS2 subtype was more sensitive to lisitinib and gefitinib (Figure 8A). A significant difference was also observed in other omics. For instance, the tumor mutation burden was higher in CS2 (Figure 8B), which may have led to poor prognoses in CS2. In addition, Figure 8C shows that CS2 had higher amounts of copy number alterations, copy number losses, and copy number-gained genomes compared to CS1 (*p* < 0.001).

### 3.11. Subtype classification verification using an external dataset

**Figure 9:**
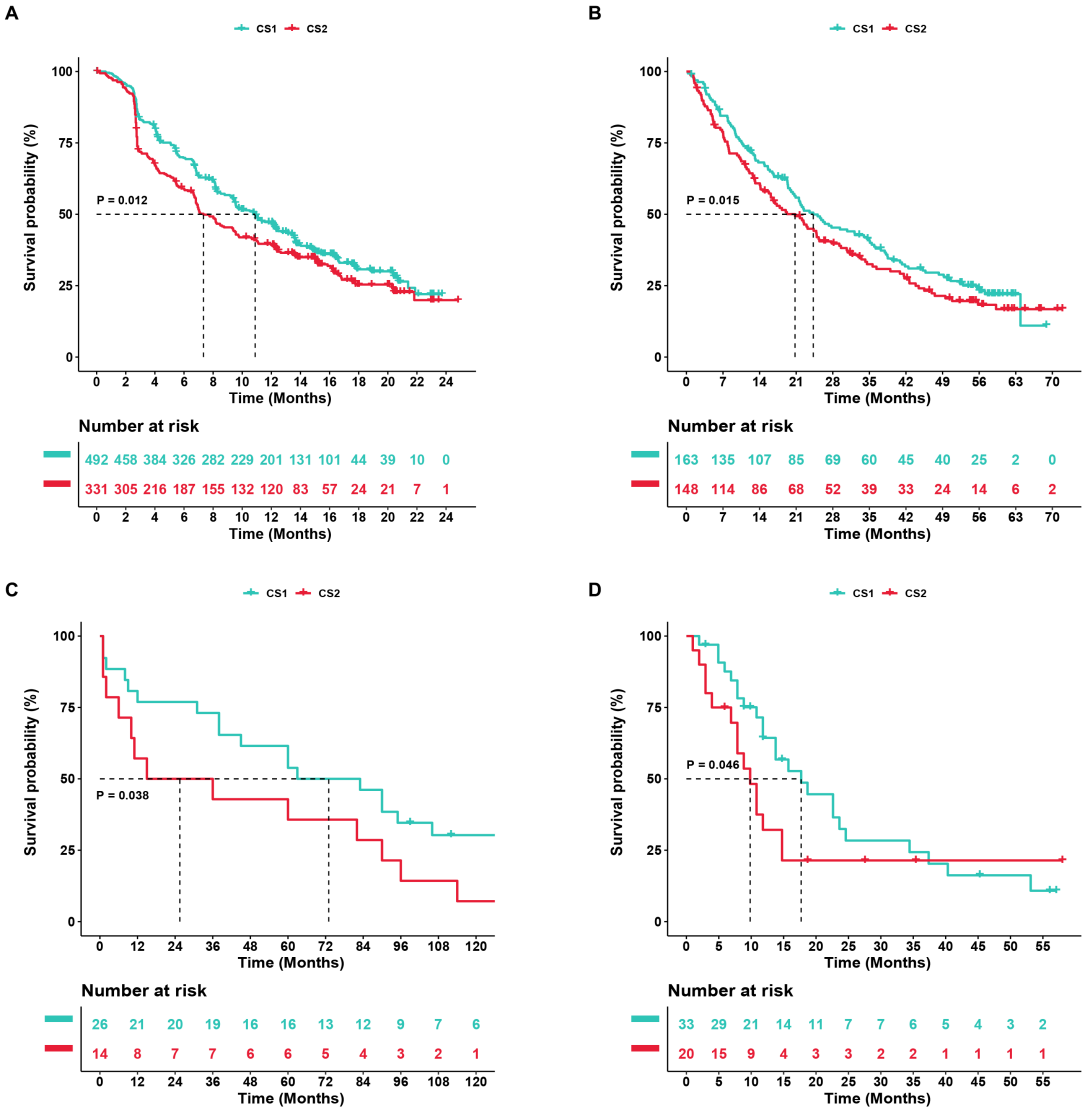
Subtype classification verification using an external dataset. **(A–D)** Kaplan–Meier curve of the NTP (nearest template prediction) by the Cancer Cell, Checkmate OS, GSE22541, and E-MTAB-3267 databases.

To further confirm the credibility of the ccRCC subtypes, the nearest template prediction algorithms were applied to reconstruct the subtypes in the TCGA–KIRC cohort and divide this cohort into two different subgroups. As shown in Figures 9A–D, the four Kaplan–Meier curves created using the data from different databases revealed that CS1 had a significantly better survival probability than CS2. In the Cancer Cell database, the value of p was 0.012; in the GSE22541 database, the value of p was 0.038; in the CheckMate OS database, the value of p was 0.015; and in the E-MTAB-3267 database, the value of p was 0.046. The stability and reliability of the subtypes were verified based on these results.

## 4. Discussion

AKI is a global concern and challenge associated with high mortality and healthcare costs worldwide (17). It can be classified into pre-renal, intrinsically renal, or post-renal types. Meanwhile, ischemia-reperfusion, a pre-renal injury, is an inevitable event in kidney transplantation surgery (18). In addition, various components of the innate and adaptive immune systems are involved in AKI pathogenesis and repair. After ischemia and reperfusion, free radical-mediated injury releases pro-inflammatory cytokines and induces innate immunity (18, 19). Following this, immune cells traverse the postischemic kidney and demonstrate changes in the associated signaling pathways, and ischemic kidney injury is also established over time (20).

Kidney function biomarkers have emerged as new tools for risk assessment and clinical therapy guidance. These biomarkers are of particular interest because several microarray studies have demonstrated differential expression of single molecules or hub genes, which are believed to play a role in the development of certain incentive-induced AKI models (21–25). In this study, we screened early-stage AKI profiles derived from renal transplant and ordinary kidney biopsy tissues from the GEO database, conducted comprehensive analyses of four GEO datasets, and obtained seven genes from four machine learning models for ischemia–reperfusion injury AKI prediction. Moreover, we integrated the identified DEGs to identify potential biomarkers and pathways involved in AKI pathogenesis. In the GO database, protein tyrosine/threonine phosphatase activity, MAP kinase tyrosine/serine/threonine phosphatase activity, and MAP kinase phosphatase activity were significant associated with AKI. Moreover, the GSEA revealed that the cytokine-cytokine receptor interaction, IL-17, and TNF signaling pathways were positively upregulated in AKI.

Numerous studies have shown that tyrosine phosphatase and MAP kinase activities have essential physiological and pathological functions in kidney diseases (26, 27). Previous studies have also suggested that IL-17 mediates renal fibrosis and neutrophil infiltration in ischemia reperfusion (28, 29). In addition, the TNF pathway was reported to participate in renal glomerular endothelial injury (30). To clarify the differential enrichment of immune cells and elucidate their possible mechanisms of action in AKI, we analyzed marker genes associated with renal immunity. Our analysis revealed significant differences in immune, stromal, and estimated scores between the AKI and normal clusters. Additionally, immune cells, including activated CD4 T cells, CD56^dim^ natural killer cells, eosinophils, mast cells, memory B cells, natural killer T cells, neutrophils, T follicular helper cells, and type 1 T helper cells were significantly upregulated in the AKI group. Consistent with previous studies, T cells are known to release proinflammatory cytokines and chemokines to promote acute renal injury, whereas natural killer T cells can induce renal inflammation and injury or protect against it, depending on the context of their activation (31, 32). Furthermore, to determine the exact AKI risk-prediction efficiency, a highly accurate predictive nomogram was constructed by integrating a seven-gene risk score. Satisfactory agreement was observed in the calibration plot of the nomogram, suggesting that our seven-gene-based risk nomogram may contribute to predicting the risk of AKI and provide early treatment guidance beyond single conventional clinical parameters.

Facilitated by bioinformatics and sequencing technology, seven genes (*EGR1*, *SOCS3*, *TRIB1*, *CCNL1*, *HBB*, *HSPA6*, and *NFKBIZ*) were identified as precise indicators of AKI. Notably, earlier studies have confirmed that these genes participate in the renal physiology and pathology of AKI. Early growth response 1 (EGR1) is an early transcription factor that is induced by many cellular factors, including growth factors and hypoxia (33). Previous studies have validated that EGR1 is associated with mediating renal epithelial cell regeneration and is significantly upregulated in AKI kidney samples (34, 35). EGR1 also regulates inflammation and fibrosis in kidney tissues (36). And upregulation of SOCS3 in stressed proximal tubules also plays an important role in AKI by modulating the macrophage phenotype and inhibiting reparative proliferation (37). TRIB1 (38) might be a therapeutic target for renal injury through the regulation of renal tubular cell proliferation. Gunther et al. reported an increase in blood-derived CCNL1 expression in kidney transplant recipients with acute rejection compared to recipients with no rejection (39). Other studies have indicated that *HBB*, *HSPA6*, and *NFKBIZ* may play essential roles in kidney disease (40–42). The roles of these genes in ccRCC also have been reported. For instance, EGR1 serves as an independent prognostic factor in ccRCC patients by inhibiting the proliferation, invasion, and metastasis of ccRCC (43). In addition, Tomita et al. found that SOCS3 might be a potential target in IFN-α-resistant RCC treatment (44). Furthermore, studies have reported that NFKBIZ and HSPA6 are associated with ccRCC (42, 45).

Inflammatory microenvironments often develop prior to malignant changes and tumorigenesis (46). A previous study indicated that AKI induces malignant renal cell carcinoma *via* CXCR2 in the proximal tubular kidney epithelial cells of mice (12). These findings highlight the potential of using these seven genes (*CCNL1*, *NFKBIZ*, *HBB*, *TRIB1*, *SOCS3*, *HSPA6*, *EGR1*) in guiding clinical decision-making and the early prevention of AKI. In order to determine if certain AKI signatures have predictive value for ccRCC, we divided the ccRCC patient data into two clusters based on the key AKI genes. We found that CS1 had better prognostic value compared to CS2 in terms of OS and PFS. In addition, the expression profile of *EGR1* was more enriched in normal tissues than in CS1 and CS2. This suggests that *EGR1* may inhibit the proliferation, invasion, and metastasis of ccRCC, which is consistent with previous results (43). *CCNL1*, *SOCS3*, and *HSPA6*, which are highly expressed in patients with ccRCC, may promote tumor generation. Immune-related signatures were highly expressed in CS2 cells, whereas immune-related inhibitors were dysregulated. Another observation was that oncogenes related to G2M-checkpoint, TGFβ signaling, and E2F targets were more enriched in CS2. The higher tumor mutation burden and fraction of genome alerts may also explain the poor prognosis of CS2. Furthermore, the CS1 subtype was more sensitive to drug therapy. Thus, this subtype classification will contribute to ccRCC prognostic analysis, the early prevention of carcinogenesis, and guidelines for clinical immunotherapy.

Machine learning, which benefits from artificial intelligence and computer science, is widely used in nephrology research (47). Chen et al. used a XGBoost model to predict progression at 5 years in patients with biopsy-proven IgA nephropathy (48). For other applications, several studies have utilized machine learning to predict the development of acute kidney injury following surgery and volume responsiveness in patients with oliguric acute kidney injury (49–51). Recently, shared gene signatures and molecular mechanisms have been demonstrated between these two diseases (52, 53). This is relevant because shared genes are important for disease prevention and early treatment. In contrast to other studies, we obtained AKI signatures using machine learning and elaborated on the value of these biomarkers for predicting AKI risk and ccRCC prognosis. However, our study does have some limitations, as most of our results were based on bioinformatics analyses. Therefore, follow-up experiments should be conducted in AKI and ccRCC, and the specific roles of these seven signatures require further investigation in future molecular experiments.

## 5. Conclusion

In conclusion, a comprehensive bioinformatics analysis of AKI was conducted, and seven genes (*CCNL1*, *NFKBIZ*, *HBB*, *TRIB1*, *SOCS3*, *HSPA6*, and *EGR1*) that may be involved in the biological processes of AKI were identified. We also explored the immune correlations associated with these seven signatures and evaluated their risk associations. Our analysis showed that CS1, along with the AKI signature cluster, may lead to better outcomes and treatment sensitivity for ccRCC. These findings provide potential targets for risk prediction and offer a new perspective on the relationship between kidney injury and kidney cancer.

## Data availability statement

The datasets presented in this study can be found in online repositories. The names of the repository/repositories and accession number(s) can be found in the article/Supplementary material.

## Author contributions

LW designed and wrote this manuscript. FP analyzed the data. ZL, MR, and YD collected the data. ZM and LL supervised the manuscript. All authors contributed to the study and approved the submitted version.

## Funding

This study was supported by the Youth Scientific Research Project of Shanghai Municipal Health Commission [grant number: 20214Y0367] and the Youth Startup Fund of Jinshan Hospital of Fudan University [grant number: JYQN-LC-202007].

## Conflict of interest

The authors declare that the research was conducted in the absence of any commercial or financial relationships that could be construed as a potential conflict of interest.

## Publisher’s note

All claims expressed in this article are solely those of the authors and do not necessarily represent those of their affiliated organizations, or those of the publisher, the editors and the reviewers. Any product that may be evaluated in this article, or claim that may be made by its manufacturer, is not guaranteed or endorsed by the publisher.

## Supplementary material

SUPPLEMENTARY FIGURE S1 Function enrichment analysis between the CS1 and CS2 subgroups. **(A)** Enhanced volcano map of the differentially expressed genes in the two clusters. **(B)** GO enrichment analysis. **(C)** GSVA analysis of the differential pathways between the two ccRCC subtypes. **(D)** Regulon scores of different transcriptional factors. Yellow represents active expression of the transcription factors. Blue represents repressed expression of the transcription factors.

SUPPLEMENTARY FIGURE S2 Immune landscapes of the two ccRCC subtypes. **(A)** Heatmap of the immune signatures between CS1 and CS2, including chemokine, chemokine receptor, MHC, immunoinhibitory, and immunostimulatory signatures. **(B)** Heatmap of tumor-infiltrating immune cells between CS1 and CS2 based on the TIMER, CIBERSORT, CIBERSORT-ABS, quanTIseq, MCP-counter, xCell, and EPIC algorithms. MHC, major histocompatibility complex.

## References

[1] Levey AS James MT. Acute kidney injury. Ann Intern Med. (2017) 167:ITC66-ITC80. doi: 10.7326/AITC201711070 29114754

[2] Coca SG Singanamala S Parikh CR. Chronic kidney disease after acute kidney injury: a systematic review and Meta-analysis. Kidney Int. (2012) 81:442–8. doi: 10.1038/ki.2011.379 22113526 PMC3788581

[3] Hoste EA Clermont G Kersten A Venkataraman R Angus DC De Bacquer D. Rifle criteria for acute kidney injury are associated with hospital mortality in critically ill patients: a cohort analysis. Crit Care. (2006) 10:R73. doi: 10.1186/cc4915 16696865 PMC1550961

[4] Oh DJ. A long journey for acute kidney injury biomarkers. Ren Fail. (2020) 42:154–65. doi: 10.1080/0886022X.2020.1721300 32050834 PMC7034110

[5] Bejnordi BE Veta M Van Diest PJ Van Ginneken B Karssemeijer N Litjens G. Diagnostic assessment of deep learning algorithms for detection of lymph node metastases in women with breast Cancer. JAMA. (2017) 318:2199–10. doi: 10.1001/jama.2017.14585 29234806 PMC5820737

[6] Lee CK Hofer I Gabel E Baldi P Cannesson M. Development and validation of a deep neural network model for prediction of postoperative in-hospital mortality. Anesthesiology. (2018) 129:649–2. doi: 10.1097/ALN.0000000000002186 29664888 PMC6148401

[7] Peired AJ Lazzeri E Guzzi F Anders HJ Romagnani P. From kidney injury to kidney Cancer. Kidney Int. (2021) 100:55–66. doi: 10.1016/j.kint.2021.03.011 33794229

[8] Peired AJ Antonelli G Angelotti ML Allinovi M Guzzi F Sisti A. Acute kidney injury promotes development of papillary renal cell adenoma and carcinoma from renal progenitor cells. Sci Transl Med. (2020) 12:eaaw6003. doi: 10.1126/scitranslmed.aaw6003 32213630

[9] Gahan JC Gosalbez M Yates T Young EE Escudero DO Chi A. Chemokine and chemokine receptor expression in kidney tumors: molecular profiling of histological subtypes and association with metastasis. J Urol. (2012) 187:827–3. doi: 10.1016/j.juro.2011.10.150 22245330 PMC5221742

[10] Eruslanov E Stoffs T Kim WJ Daurkin I Gilbert SM Su LM. Expansion of Ccr8(+) inflammatory myeloid cells in Cancer patients with Urothelial and renal carcinomas. Clin Cancer Res. (2013) 19:1670–80. doi: 10.1158/1078-0432.CCR-12-2091 23363815 PMC3618575

[11] de Vivar Chevez AR Finke J Bukowski R. The role of inflammation in kidney Cancer. Adv Exp Med Biol. (2014) 816:197–4. doi: 10.1007/978-3-0348-0837-8_9 24818725

[12] Zhou X Xiao F Sugimoto H Li B McAndrews KM Kalluri R. Acute kidney injury instigates malignant renal cell carcinoma via Cxcr2 in mice with inactivated Trp53 and Pten in proximal tubular kidney epithelial cells. Cancer Res. (2021) 81:2690–02. doi: 10.1158/0008-5472.CAN-20-2930 33558337 PMC12048857

[13] Lowrance WT Ordoñez J Udaltsova N Russo P Go AS. Ckd and the risk of incident Cancer. J Am Soc Nephrol. (2014) 25:2327–34. doi: 10.1681/ASN.2013060604 24876115 PMC4178430

[14] Hsieh JJ Purdue MP Signoretti S Swanton C Albiges L Schmidinger M. Renal Cell Carcinoma. Nat Rev Dis Primers. (2017) 3:1–19. doi: 10.1038/nrdp.2017.9 PMC5936048 28276433

[15] Jiang A Pang Q Gan X Wang A Wu Z Liu B. Definition and verification of novel metastasis and recurrence related signatures of Ccrcc: a multicohort study. Cancer Innovation. (2022) 1:146–7. doi: 10.1002/cai2.25 PMC10686128 38090653

[16] Jiang A Wu X Wang D Wang A Dong K Liu B. A new thinking: deciphering the aberrance and clinical implication of Igf Axis regulation pattern in clear cell renal cell carcinoma. Front Immunol. (2022) 13:935595. doi: 10.3389/fimmu.2022.935595 35935986 PMC9355597

[17] Lameire NH Bagga A Cruz D De Maeseneer J Endre Z Kellum JA. Acute kidney injury: An increasing global concern. Lancet. (2013) 382:170–9. doi: 10.1016/S0140-6736(13)60647-9 23727171

[18] Kosieradzki M Rowinski W. Ischemia/reperfusion injury in kidney transplantation: mechanisms and prevention. Transplant Proc. (2008) 40:3279–88. doi: 10.1016/j.transproceed.2008.10.004 19100373

[19] Jang HR Rabb H. The innate immune response in ischemic acute kidney injury. Clin Immunol. (2009) 130:41–50. doi: 10.1016/j.clim.2008.08.016 18922742 PMC2646108

[20] Zheng L Gao W Hu C Yang C Rong R. Immune cells in ischemic acute kidney injury. Curr Protein Pept Sci. (2019) 20:770–6. doi: 10.2174/1389203720666190507102529 31060484

[21] Yang JJ Wu BB Han F Chen JH Yang Y. Gene expression profiling of Sepsis-associated acute kidney injury. Exp Ther Med. (2020) 20:34. doi: 10.3892/etm.2020.9161 32952625 PMC7485311

[22] Tang Y Yang X Shu H Yu Y Pan S Xu J. Bioinformatic analysis identifies potential biomarkers and therapeutic targets of septic-shock-associated acute kidney injury. Hereditas. (2021) 158:13. doi: 10.1186/s41065-021-00176-y 33863396 PMC8052759

[23] Lin X Li J Tan R Zhong X Yang J Wang L. Identification of hub genes associated with the development of acute kidney injury by weighted gene co-expression network analysis. Kidney Blood Press Res. (2021) 46:63–73. doi: 10.1159/000511661 33401265

[24] Wei J Zhang J Wei J Hu M Chen X Qin X. Identification of Agxt2, Shmt1, and Aco2 as important biomarkers of acute kidney injury by Wgcna. PLoS One. (2023) 18:e0281439. doi: 10.1371/journal.pone.0281439 36735737 PMC9897545

[25] Kagawa T Zarybnicky T Omi T Shirai Y Toyokuni S Oda S. A scrutiny of circulating Microrna biomarkers for drug-induced tubular and glomerular injury in rats. Toxicology. (2019) 415:26–36. doi: 10.1016/j.tox.2019.01.011 30682439

[26] Jung YJ Park W Kang KP Kim WJNDT. Sirt2 is involved in Cisplatin-induced acute kidney injury through regulation of mitogen-activated protein kinase Phosphatase-1. Nephrol Dialysis Transpl. (2020) 35:1145–56. doi: 10.1093/ndt/gfaa042 32240312

[27] Li H Xiong J Du Y Huang Y Zhao J. Dual-specificity phosphatases and kidney diseases. Kidney Dis (Basel). (2022) 8:13–25. doi: 10.1159/000520142 35224004 PMC8820169

[28] Li L Huang L Vergis AL Ye H Bajwa A Narayan V. Il-17 produced by neutrophils regulates Ifn-gamma-mediated neutrophil migration in mouse kidney ischemia-reperfusion injury. J Clin Invest. (2010) 120:331–2. doi: 10.1172/JCI38702 20038794 PMC2798679

[29] Mehrotra P Collett JA McKinney SD Stevens J Ivancic CM Basile DP. Il-17 mediates neutrophil infiltration and renal fibrosis following recovery from ischemia reperfusion: compensatory role of natural killer cells in Athymic rats. Am J Physiol Renal Physiol. (2017) 312:F385–97. doi: 10.1152/ajprenal.00462.2016 27852609 PMC5374313

[30] Xu C Chang A Hack BK Eadon MT Alper SL Cunningham PN. Tnf-mediated damage to glomerular endothelium is an important determinant of acute kidney injury in Sepsis. Kidney Int. (2014) 85:72–81. doi: 10.1038/ki.2013.286 23903370 PMC3834073

[31] Kinsey GR Okusa MD. Expanding role of T cells in acute kidney injury. Curr Opin Nephrol Hypertens. (2014) 23:9–16. doi: 10.1097/01.mnh.0000436695.29173.de 24231312 PMC3909711

[32] Jang HR Rabb H. Immune cells in experimental acute kidney injury. Nat Rev Nephrol. (2015) 11:88–1. doi: 10.1038/nrneph.2014.180 25331787

[33] Huh J-E Nam D-W Baek Y-H Kang JW Park D-S Choi D-Y. Formononetin accelerates wound repair by the regulation of early growth response Factor-1 transcription factor through the phosphorylation of the Erk and P38 Mapk pathways. Int Immunopharmacol. (2011) 11:46–54. doi: 10.1016/j.intimp.2010.10.003 20959155

[34] Chen J Chen Y Olivero A Chen XJ. Identification and validation of potential biomarkers and their functions in acute kidney injury. Front Genet. (2020) 11:1. doi: 10.3389/fgene.2020.00411 32528518 PMC7247857

[35] Chen JW Huang MJ Chen XN Wu LL Li QG Hong Q. Transient Upregulation of Egr1 signaling enhances kidney repair by activating Sox9(+) renal tubular cells. Theranostics. (2022) 12:5434–50. doi: 10.7150/thno.73426 35910788 PMC9330523

[36] Ho LC Sung JM Shen YT Jheng HF Chen SH Tsai PJ. Egr-1 deficiency protects from renal inflammation and fibrosis. J Mol Med (Berl). (2016) 94:933–2. doi: 10.1007/s00109-016-1403-6 26960759

[37] Susnik N Sorensen-Zender I Rong S von Vietinghoff S Lu X Rubera I. Ablation of proximal tubular suppressor of cytokine signaling 3 enhances tubular cell cycling and modifies macrophage phenotype during acute kidney injury. Kidney Int. (2014) 85:1357–68. doi: 10.1038/ki.2013.525 24402091

[38] Xie X Yang X Wu J Ma J Wei W Fei X. Trib1 contributes to recovery from ischemia/reperfusion-induced acute kidney injury by regulating the polarization of renal macrophages. Front Immunol. (2020) 11:473. doi: 10.3389/fimmu.2020.00473 32265926 PMC7098949

[39] Gunther OP Shin H Ng RT McMaster WR McManus BM Keown PA. Novel multivariate methods for integration of genomics and proteomics data: applications in a kidney transplant rejection study. OMICS. (2014) 18:682–5. doi: 10.1089/omi.2014.0062 25387159 PMC4229708

[40] Liu Z Tang C He L Yang D Cai J Zhu J. The negative feedback loop of Nf-Kappab/Mir-376b/Nfkbiz in septic acute kidney injury. JCI. Insight. (2020) 5:1–17. doi: 10.1172/jci.insight.142272 PMC7819752 33328388

[41] Naik RP Derebail VK Grams ME Franceschini N Auer PL Peloso GM. Association of Sickle Cell Trait with chronic kidney disease and albuminuria in African Americans. JAMA. (2014) 312:2115–25. doi: 10.1001/jama.2014.15063 25393378 PMC4356116

[42] Yang ES Nassar AH Adib E Jegede OA Alaiwi SA Manna DLD. Gene expression signature correlates with outcomes in metastatic renal cell carcinoma patients treated with Everolimus alone or with a vascular disrupting Agentgene signature correlates with outcomes for metastatic Rcc. Mol Cancer Ther. (2021) 20:1454–61. doi: 10.1158/1535-7163.MCT-20-1091 34108261

[43] Zhang ZY Zhang SL Chen HL Mao YQ Kong CY Li ZM. Low Egr1 expression predicts poor prognosis in clear cell renal cell carcinoma. Pathol Res Pract. (2021) 228:153666. doi: 10.1016/j.prp.2021.153666 34749216

[44] Tomita S Ishibashi K Hashimoto K Sugino T Yanagida T Kushida N. Suppression of Socs3 increases susceptibility of renal cell carcinoma to interferon-alpha. Cancer Sci. (2011) 102:57–63. doi: 10.1111/j.1349-7006.2010.01751.x 21054677 PMC11159134

[45] Hao J Cao Y Yu H Zong L An R Xue Y. Effect of Map3k8 on prognosis and tumor-related inflammation in renal clear cell carcinoma. Front Genet. (2021) 12:674613. doi: 10.3389/fgene.2021.674613 34567061 PMC8461076

[46] Mantovani A Allavena P Sica A Balkwill F. Cancer-related inflammation. Nature. (2008) 454:436–4. doi: 10.1038/nature07205 18650914

[47] Jordan MI Mitchell TM. Machine learning: trends, perspectives, and prospects. Science. (2015) 349:255–14. doi: 10.1126/science.aaa8415 26185243

[48] Chen T Li X Li Y Xia E Qin Y Liang S. Prediction and risk stratification of kidney outcomes in Iga nephropathy. Am J Kidney Dis. (2019) 74:300–9. doi: 10.1053/j.ajkd.2019.02.016 31031086

[49] Tseng PY Chen YT Wang CH Chiu KM Peng YS Hsu SP. Prediction of the development of acute kidney injury following cardiac surgery by machine learning. Crit Care. (2020) 24:478. doi: 10.1186/s13054-020-03179-9 32736589 PMC7395374

[50] Zhang Z Ho KM Hong Y. Machine learning for the prediction of volume responsiveness in patients with Oliguric acute kidney injury in critical care. Crit Care. (2019) 23:112. doi: 10.1186/s13054-019-2411-z 30961662 PMC6454725

[51] Mohamadlou H Lynn-Palevsky A Barton C Chettipally U Shieh L Calvert J. Prediction of acute kidney injury with a machine learning algorithm using electronic health record data. Can J Kidney Health Dis. (2018) 5:776326. doi: 10.1177/2054358118776326 PMC6080076 30094049

[52] Liang Z Hu X Lin R Tang Z Ye Z Mao R. Identification of shared gene signatures and molecular mechanisms between chronic kidney disease and ulcerative colitis. Front Immunol. (2023) 14:1078310. doi: 10.3389/fimmu.2023.1078310 36860851 PMC9970095

[53] Yao M Zhang C Gao C Wang Q Dai M Yue R. Exploration of the shared gene signatures and molecular mechanisms between systemic lupus Erythematosus and pulmonary arterial hypertension: evidence from Transcriptome data. Front Immunol. (2021) 12:658341. doi: 10.3389/fimmu.2021.658341 34335565 PMC8320323

