# Supplementary figures and images for: Identification of AKI signatures and classification patterns in ccRCC based on machine learning
Source: Front Med (Lausanne). 2023 May 24;10:1195678. doi: 10.3389/fmed.2023.1195678 (PMC10244623; doi:10.3389/fmed.2023.1195678)

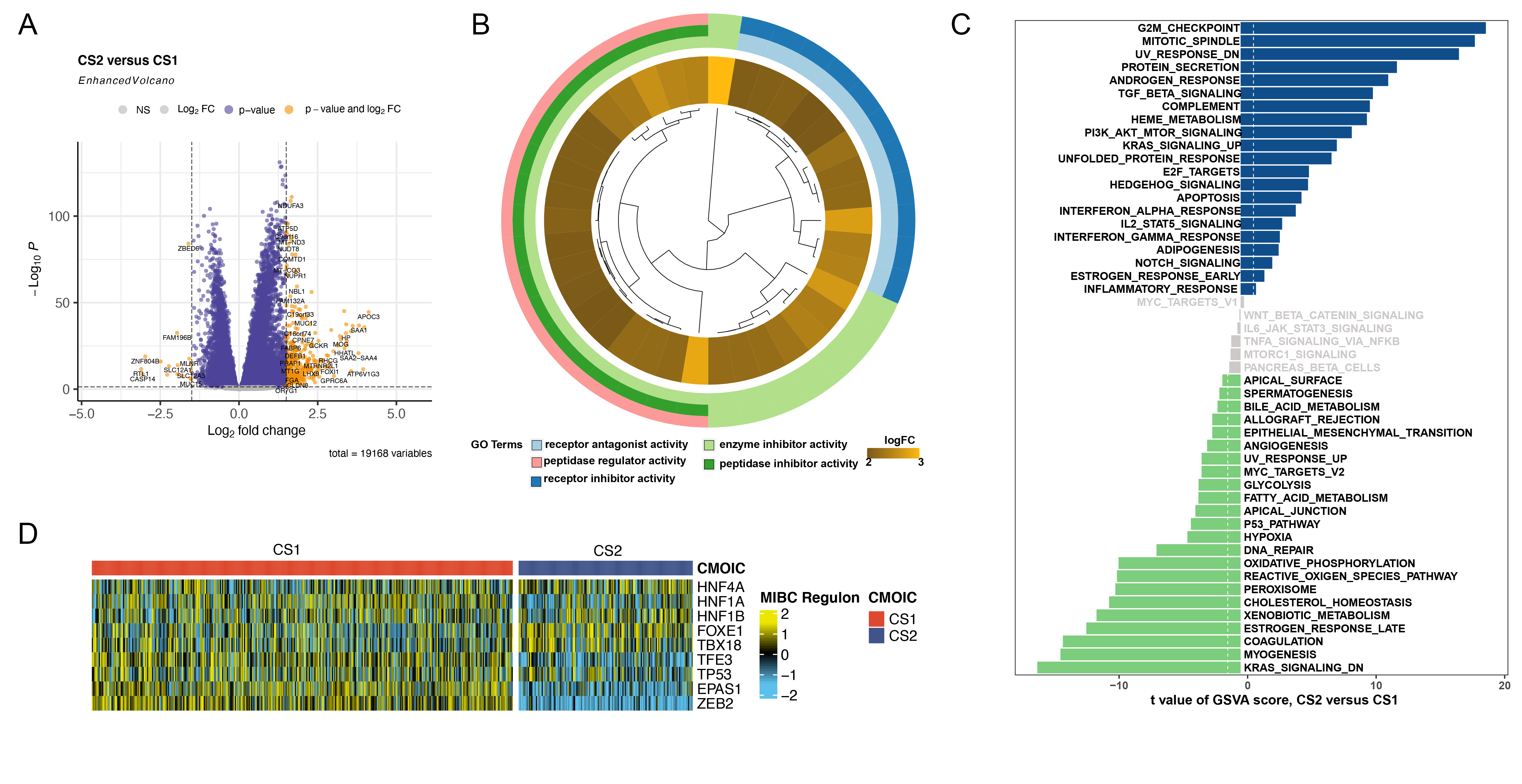

Supplement: SUPPLEMENTARY FIGURE S1 — Function enrichment analysis between the CS1 and CS2 subgroups. (A) Enhanced volcano map of the differentially expressed genes in the two clusters. (B) GO enrichment analysis. (C) GSVA analysis of the differential pathways between the two ccRCC subtypes. (D) Regulon scores of different transcriptional factors. Yellow represents active expression of the transcription factors. Blue represents repressed expression of the transcription factors. [file Image_1.TIF]

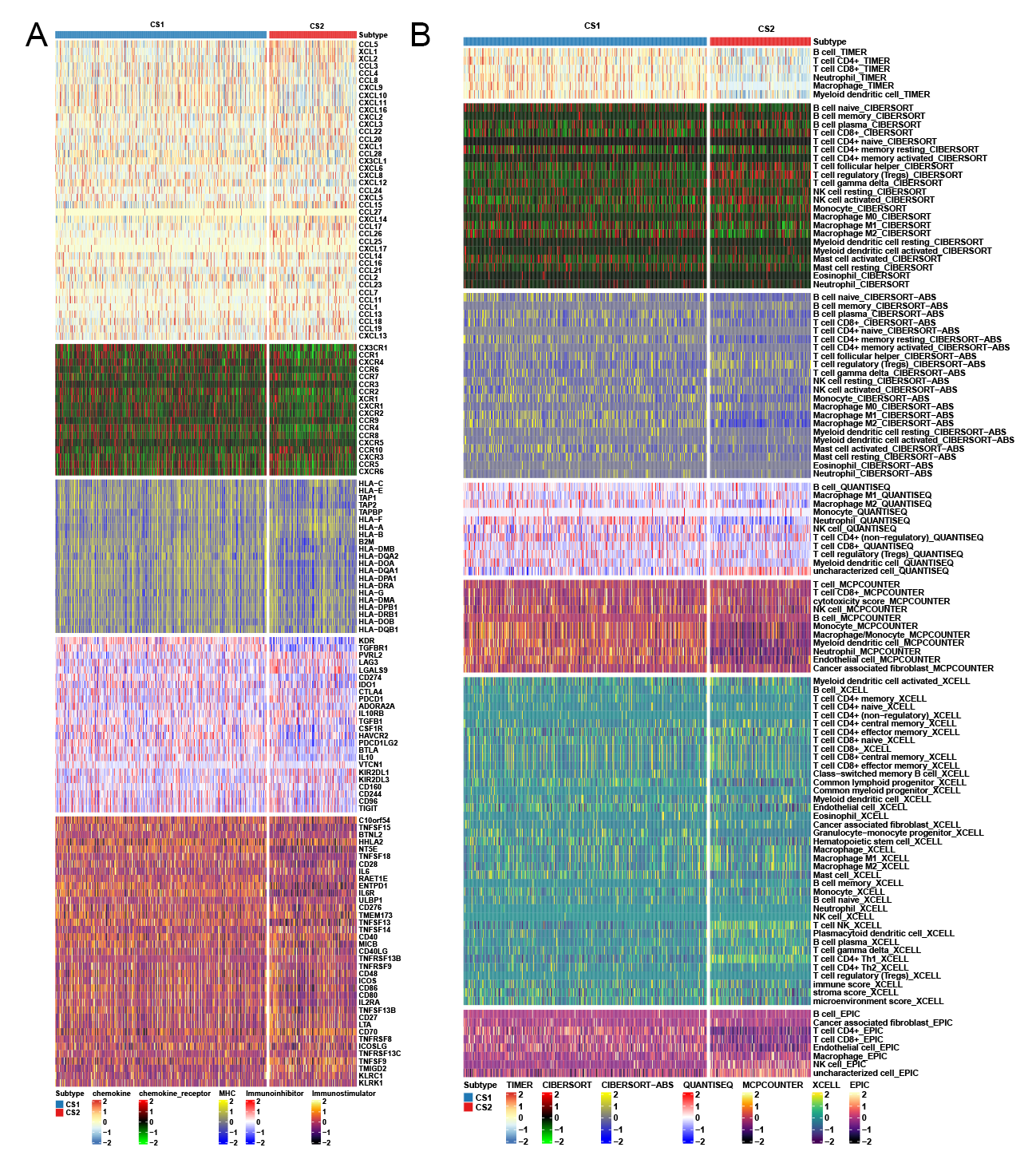

Supplement: SUPPLEMENTARY FIGURE S2 — Immune landscapes of the two ccRCC subtypes. (A) Heatmap of the immune signatures between CS1 and CS2, including chemokine, chemokine receptor, MHC, immunoinhibitory, and immunostimulatory signatures. (B) Heatmap of tumor-infiltrating immune cells between CS1 and CS2 based on the TIMER, CIBERSORT, CIBERSORT-ABS, quanTIseq, MCP-counter, xCell, and EPIC algorithms. MHC, major histocompatibility complex. [file Image_2.TIF]
